# Supplementary figures and images for: Changes in physical activity, dietary and sleeping pattern among the general population in COVID-19: A systematic review protocol
Source: PLoS One. 2022 Jun 3;17(6):e0269202. doi: 10.1371/journal.pone.0269202 (PMC9165901; doi:10.1371/journal.pone.0269202)

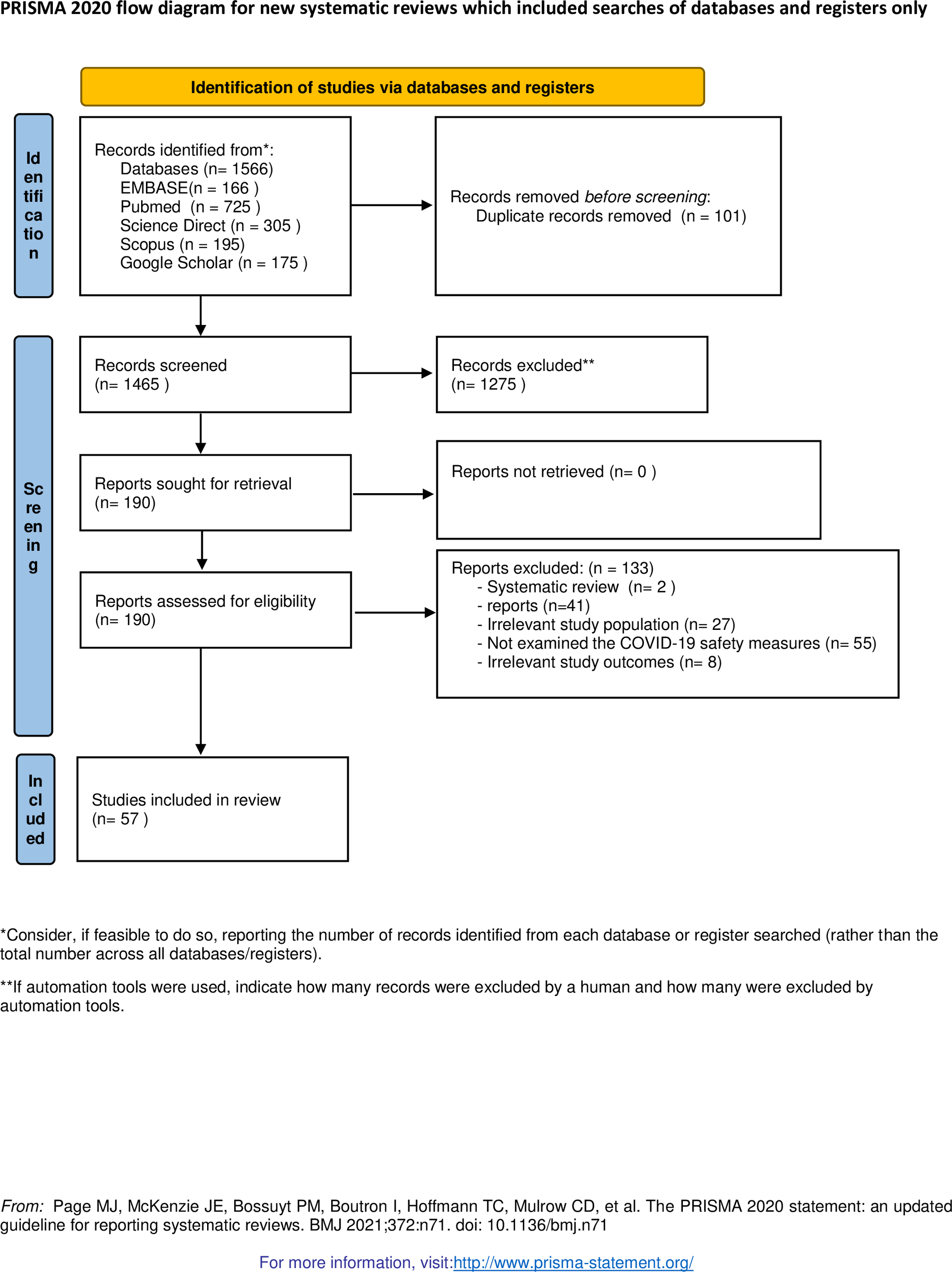

Supplement: S2 File — (TIF) [file pone.0269202.s002.tif]
