# Supplementary material for: Changes in physical activity, dietary and sleeping pattern among the general population in COVID-19: A systematic review protocol
Source: PLoS One. 2022 Jun 3;17(6):e0269202. doi: 10.1371/journal.pone.0269202 (PMC9165901; doi:10.1371/journal.pone.0269202)
Supplement: Attachment — Submitted filename: Respnose to reviewers.docx [file pone.0269202.s003.docx]

**List of Amendments**

Line number- 24 (Abstract)

"Were" was corrected by "will be" because it should be future tense.

Line number- 25 (Abstract)

"applied and search work will take one month" were added to clearly mention the planned time of search work.

Line number- 26 (Abstract)

"usages" was substituted by "characteristics" because key search terms include the characteristics of physical activity (e.g. frequency, duration), diet (e.g. patterns, food types) and sleep (quality, timing).

Line number- 28 and 29 (Abstract)

" as well as the screening form and the PRISMA flow" were added because these are important information for the selection process of this review.

Line number-55 (Introduction)

Citation [5] was moved to the end of the relevant information in line numbers- 53 to 55 and put together with citations [6, 7, 8] (Line number-55) because these discussion points were concerned with citation [5].

Line number- 58 (Introduction)

Citation [8] was moved to the end of the relevant information and put together with citation [9] (Line number-58) because citation [8] covered the information of line numbers- 55 to 58.

Line number- 67 (Introduction)

Citations [13, 14] were removed due to duplication and putting them [13,14] (Line number-67) at the end of the summarized or paraphrased information will be more relevant.

Line number- 69 (Introduction)

Citation [15] was removed due to duplication.

Line number- 75 (Introduction)

Citation [4] was removed because the suggestion points cited with [4] and [16] were more relevant to only citation [16].

Line number- 76 (Last paragraph of the Introduction Section)

The last paragraph of the introduction section was substituted by some information in order to be the relevant and strong justification of the review objective.

Line number- 99 (Methods)

"a" was added for grammar correctness.

Line number- 102 (Methods)

"will be totally applied" were added for grammar correctness.

Line number- 105 (Eligibility Criteria)

"the effects of preventive public health measures" were substituted by "the Covid-19 restrictions" which are more precise words.

Line number- 118-121 (Controls)

The paragraph under the sub-title of "Controls" was removed and restructured with " This review will not try to analyze the statistical significances of the changing effects of physical activity, diet and sleep between healthy and unhealthy groups. Therefore, control studies and comparisons between the general population and other control groups will not be considered to include in this review."

Line number- 133 (List and Definitions of Variables)

"sex" was corrected by "sexes" for grammar correctness.

Line number- 158-160 (Study Design)

"because this review wants to explore the events of many countries affected by the Covid-19 and the Covid-19 widely spread across the world after October 2019" were added for justifying the publication date eligible for this review.

Line number- 168-175 (Information Sources and Search Strategy)

"Developing the screening form (including search terms, number of references screened per time, per reviewer and per database, reasons for inclusion/exclusion), exercising independent searches, discussions among reviewers will be ensured. Besides, inter-reviewer agreement form (including "Paper Code", "Paper Title", "Includable-Yes", "Excludable-No", Uncertain-Maybe" and "Reasons for Inclusion/Exclusion") will be developed and practiced for systematic selection of the studies. The completed search will take about one month and therefore will be planned to start on January 8, 2021, and end on February 8, 2021." were added for providing more information of search and selection processes.

Line number-179 (Table.1. Search Strategy)

"Duration (PA), Frequency (PA), Intensity (PA) and Types (PA)" were added under the column of Physical Activity for application of detailed search.

Line number- 216 (Limitations of the study design)

"bias" was corrected by "biases" for grammar correctness.

**PRISMA checklist**

In the connection with previous email, we uploaded the “PRISMA checklist” in this time. However, we had already uploaded the PRISMA-P checklist (S1 file) and I think it is entitled for the study protocol. May I upload both files if you don’t mind.
