# Supplementary material for: Changes in physical activity, dietary and sleeping pattern among the general population in COVID-19: A systematic review protocol
Source: PLoS One. 2022 Jun 3;17(6):e0269202. doi: 10.1371/journal.pone.0269202 (PMC9165901; doi:10.1371/journal.pone.0269202)
Supplement: Attachment — Submitted filename: Response to reviewers.docx [file pone.0269202.s004.docx]

**List of Amendments**

1. Line 49-51 (Introduction)

“that can transform the different forms of variants: Alpha-B.1.1.7, Beta-B.1.351, Gamma-P.1, Delta-B.1.617.2 and Omicron-BA.1 or BA.2 [1]. Its infectivity“ was added and Citation [1] was newly added.

2. Line 52 (Introduction)

Citation number was changed from [1-3] to [2-4].

3. Line 54 (Introduction)

Citation number was changed from [4] to [5].

4. Line 54-57 (Introduction)

"This mandatory self-isolation may affect people’s physical and mental health" were was substituted by "since lifting the strategic restrictions has some important advantages such as limiting the spread of the Covid-19 virus, enhancing more preparation time for the preventive strategies and avoiding the new peak of the Covid019 outbreak [6]. However, these restriction measures are mandatory". Citation number [6] was newly added.

5. Line 59 (Introduction) (Reviewer #1’s comment)

Citation number was changed from [5-8] to [7-12] and citation number [11] and [12] was newly added to provide evidence for relevant information.

6. Line number- 60 (Introduction)

"may give rise" was changed to "gave rise" for correction.

7. Line number- 61 (Introduction)

"the physically active people may decrease their activity" was changed to "the physically active people decreased their activity " for correction.

8. Line number- 61-62 (Introduction)

"may not be likely to increase their daily PA" was changed to "were not likely to increase their daily PA" for correction.

9. Line number- 62 (Introduction)

"prolonged homestay may decrease the amount of daily PA performed" was changed to " prolonged homestay decreased the amount of daily PA performed " for correction.

10. Line 62 (Introduction)

Citation number was changed from [8,9] to [10,13].

11. Line 63 (Introduction)

Citation number was changed from [10] to [14].

12. Line 65 (Introduction)

Citation number was changed from [11] to [15].

13. Line 67 (Introduction)

Citation number was changed from [4,12] to [5,16].

14. Line 71 (Introduction)

Citation number was changed from [13,14] to [17,18].

15. Line 74 (Introduction)

Citation number was changed from [15] to [19].

16. Line 74 - 76 (Introduction) (Reviewer #1’s comment)

‘The studies revealed that PA decreased although eating pattern was healthy [11] while those who adopted unhealthy lifestyle encountered the lower sleep quality [12].’ was newly added and the citation number [11] and [12] was also newly cited.

17. Line 78 (Introduction)

Citation number was changed from [13] to [17].

18. Line 80 (Introduction)

Citation number was changed from [4] to [5].

19. Line 81 (Introduction)

Citation number was changed from [16] to [20].

20. Line 89 (Introduction)

Citation number was changed from [17] to [21].

21. Line 122 (Materials and Methods)

Citation number was changed from [3,17] to [4,21].

22. Line 131 (Materials and Methods)

Citation number was changed from [18] to [22].

23. Line 131 (Materials and Methods)

Citation number was changed from [19] to [23].

24. Line 204 (Risk of bias (quality) assessment)

Citation number was changed from [20] to [24].

25. Line number- 248-249 (References)

The following reference was added.

1. Genomic Epidemiology of hCov-19 [Internet]. GISAID - Initiative. 2022 [cited 2022 Apr 30]. Available from: <https://www.gisaid.org/>
26. Line number- 263-266 (References)

The following reference was added.

6. Coronavirus disease (COVID-19): Herd immunity, lockdowns and COVID-19 [Internet]. WHO | World Health Organization. [cited 2022 Apr 30]. Available from: https://www.who.int/news-room/questions-and-answers/item/herd-immunity-lockdowns-and-covid-19

27. According to reviewer #1, Durucan et al. 2022 [10.1097/MRR.0000000000000519]; Cunha et al. 2021 [10.1016/j.numecd.2021.12.019]; Carotenuto et al. 2021 [10.3390/jcm10061234]; Beydoun et al. 2022 [10.1093/gerona/glac028]; Bruno et al. 2022 [10.1016/j.sleep.2022.01.002] were suggested to investigate how COVID-19-related restrictions impacted on sleep quality, physical exercise and dietary pattern.

We had cited both Cunha et al. 2021 [10.1016/j.numecd.2021.12.019] and Bruno et al. 2022 [10.1016/j.sleep.2022.01.002] in the manuscript, however, the rest articles were left with the reason of unaligned study population (patients).
